# Are the costs of 3D printing for surgical procedures yet to be definitively assessed?

**DOI:** 10.1186/s10195-024-00783-1

**Published:** 2024-10-12

**Authors:** Ranran Li, Sitian Niu, Jingzhi Wang

**Affiliations:** Jining Medical University, Jining, 272067 Shandong China

Dear Editor,

We deeply studied and discussed a paper “Three-dimensional printed models can reduce costs and surgical time for complex proximal humeral fractures: preoperative planning, patient satisfaction, and improved resident skills” by Fidanza et al. [1]. The authors believe that a three-dimensional (3D) printed device has great advantages in the treatment of proximal humeral fractures (PHFs). The use of 3D printing in the treatment of PHFs can reduce the frequency of intraoperative fluoroscopy and shorten the operation time, thereby reducing the risk of radiation exposure for patients and medical staff and improving the treatment effect. We are very much looking forward to the widespread use of 3D printing in the field of surgery, so we have the following comments to discuss with the authors.

First, Chen et al. [2] evaluated the loss of neck-shaft angle (NSA) and humeral head height (HHH) in patients with PHFs by postoperative imaging results. The loss of NSA and HHH is an important objective index to evaluate the postoperative joint function of patients. This study did not systematically follow up the postoperative imaging data of patients with PHFs. Evaluation of postoperative imaging results can fully reflect the value of 3D printing in the surgical treatment of complex proximal humeral fractures. Second, Monticone et al. [3] used the Short Form Health Survey (SF-36) to evaluate the degree of pain and quality of life of patients with PHFs after surgery. This study did not analyze the effect of 3D printing on postoperative pain and quality of life of patients with PHFs, which may affect the integrity of the research results to a certain extent. In addition, Hu et al. [4] found that 3D-printed treatment of PHFs would increase the economic cost of patients. However, the authors of this paper believe that 3D printing for the treatment of PHFs can save an average of 400 euros per operation. 3D printing technology requires specific equipment and high proprietary technology, as well as regular equipment maintenance and maintenance [5]. Two of the medical institutions included in this study have proprietary 3D printing equipment, which can reduce the financial cost of patients to a certain extent. Different medical institutions received different 3D printing technology and financial support. Therefore, the findings of this study lack generalizability in medical areas with poor equipment and technical support.

Once again, we thank the authors for their contributions to this study and look forward to their responses to our questions.

## Data Availability

Not applicable.
